# Development and application of an economic model (EQUIPTMOD) to assess the impact of smoking cessation

**DOI:** 10.1111/add.14001

**Published:** 2017-09-14

**Authors:** Kathryn Coyle, Doug Coyle, Adam Lester‐George, Robert West, Bertalan Nemeth, Mickael Hiligsmann, Marta Trapero‐Bertran, Reiner Leidl, Subhash Pokhrel, on behalf of the EQUIPT Study Group

**Affiliations:** ^1^ Health Economics Research Group, Institute of Environment, Health and Societies Brunel University London London UK; ^2^ School of Epidemiology, Public Health and Preventative Medicine University of Ottawa Ottawa ON Canada; ^3^ LeLan Solutions Bristol UK; ^4^ Department of Epidemiology and Public Health University College London London UK; ^5^ Syreon Research Institute Budapest Hungary; ^6^ Department of Health Services Research, School for Public Health and Primary Care (CAPHRI) Maastricht University Maastricht the Netherlands; ^7^ Centre of Research in Economics and Health (CRES‐UPF) University Pompeu Fabra Barcelona Spain; ^8^ Faculty of Economics and Social Sciences Universitat Internacional de Catalunya (UIC) Barcelona Spain; ^9^ Institute of Health Economics and Health Care Management, Helmholtz Zentrum München (GmbH)—German Research Center for Environmental Health, Comprehensive Pneumology Center Munich (CPC‐M) Member of the German Center for Lung Research (DZL) Neuherberg Germany; ^10^ Munich Center of Health Sciences Ludwig‐Maximilians‐University Munich Germany

**Keywords:** Cost‐effectiveness, Markov model, return on investment, smoking cessation, tobacco, tobacco control

## Abstract

**Background and Aims:**

Although clear benefits are associated with reducing smoking, there is increasing pressure on public health providers to justify investment in tobacco control measures. Decision‐makers need tools to assess the Return on Investment (ROI)/cost‐effectiveness of programmes. The EQUIPT project adapted an ROI tool for England to four European countries (Germany, the Netherlands, Spain and Hungary). EQUIPTMOD, the economic model at the core of the ROI tool, is designed to assess the efficiency of packages of smoking cessation interventions. The objective of this paper is to describe the methods for EQUIPTMOD and identify key outcomes associated with continued and cessation of smoking.

**Methods:**

EQUIPTMOD uses a Markov model to estimate life‐time costs, quality‐adjusted life years (QALYs) and life years associated with a current and former smoker. It uses population data on smoking prevalence, disease prevalence, mortality and the impact of smoking combined with associated costs and utility effects of disease. To illustrate the tool's potential, costs, QALYs and life expectancy were estimated for the average current smoker for five countries based on the assumptions that they continue and that they cease smoking over the next 12 months. Costs and effects were discounted at country‐specific rates.

**Results:**

For illustration, over a life‐time horizon, not quitting smoking within the next 12 months in England will reduce life expectancy by 0.66, reduce QALYs by 1.09 and result in £4961 higher disease‐related health care costs than if the smoker ceased smoking in the next 12 months. For all age–sex categories, costs were lower and QALYs higher for those who quit smoking in the 12 months than those who continued.

**Conclusions:**

EQUIPTMOD facilitates assessment of the cost effectiveness of smoking cessation strategies. The demonstrated results indicate large potential benefits from smoking cessation at both an individual and population level.

## Introduction

Smoking has a significant negative health impact, with more than 1 billion dollars spent every year on treating smoking‐attributable diseases within Europe 1. It is linked to many health conditions, including coronary heart disease, stroke, respiratory illnesses and certain cancers 2. Moreover, it is the largest avoidable risk factor, responsible for more than 700 000 European deaths each year 3. The large health and economic impact of smoking has led to recommendations to focus on reducing smoking rates and increasing measures to decrease the impact of second‐hand smoke. Although there are clear benefits to public health measures to reduce smoking, in the current era of scarce resources there is also increasing pressure on public health providers to justify the investment in tobacco control measures. Decision‐makers at regional and national levels need tools to help assess the return on investment/cost–effectiveness of programmes and to produce easily interpreted outputs, thereby allowing clear presentation of the findings as justification for implementation of smoking cessation programme proposals.

Return on Investment (ROI) forecasting models have been used in the past to help employers justify investment in health promotion programmes within a number of US companies 4. A recent example of the implementation of a novel ROI tool designed for use by local public health decision‐makers is the NICE (The National Institute for Health and Care Excellence) smoking cessation ROI tool developed for England, whose aim was to assist local decision‐makers with calculating the balance of the investment required for implementation of smoking cessation programmes and the health and economic benefits of stopping smoking 5. The European Study on Quantifying Utility of Investment in Protection from Tobacco (EQUIPT) project has been funded to build on the success of the NICE ROI 6. EQUIPT aimed to adapt the existing ROI tool for four additional core European countries (Germany, the Netherlands, Spain and Hungary) in addition to England within its initial phase, and subsequently explore the potential for these models to be adapted to meet the needs of additional European countries.

The EQUIPT ROI tool was designed to address the decision problem relating to identifying what is the optimal package of smoking cessation interventions for commissioners at all levels of smoking cessation interventions. At the core of the EQUIPT ROI tool is an economic model, EQUIPTMOD, which forecasts the short‐ and long‐term outcomes for smokers who cease smoking within the next calendar year or continue smoking. These outcomes can then be used to forecast the impact of a variety of smoking cessation interventions. The objective of this paper is to describe the methods for EQUIPTMOD and to apply the model to identify the key outcomes associated with both continued and cessation of smoking within the five initial partner countries.

## Methods

### Decision problem

EQUIPTMOD is designed to facilitate the determination of an optimal package of smoking cessation interventions. Thus, the specific decision problem relates to identifying those interventions which can be considered cost‐effective. EQUIPTMOD allows the determination of a range of relevant outcomes which are a factor of the estimated costs and outcomes associated with continuing and stopping smoking. In this paper, we will describe the methods for EQUIPTMOD and illustrate the potential of the model by estimating the life‐time costs, quality‐adjusted life years (QALYs) and life years associated with a current smoker and former smoker.

### Population

The population of interest is the current smoking population within the five core European countries. The population is stratified by age (by individual birth year) and sex (male, female), with estimates weighted by the actual number of smokers in each strata.

### Comparators

Decision‐makers can consider a variety of potential packages of smoking cessation interventions at both the individual and population levels. Three packages can be considered at any one time: default data relating to the currently implemented tobacco control intervention package within the selected country or region, a minimal investment package which relates to no continued funding of interventions and a user‐defined package. For the latter package, users can customize the investment in existing smoking cessation interventions and can incorporate new previously unfunded interventions into the package. Within the interface, users can change default parameters such as discount rates and threshold values for a QALY. Once the packages have been developed, users can overview the different potential metrics for each permissible time horizon.

### Outcomes

The flexibility of EQUIPTMOD allows the derivation of a number of metrics for determining the return on investment from the alternate packages. Metrics include avoidable burden of disease (QALYs), benefit–cost ratios, net benefit analysis and incremental cost‐effectiveness ratios incorporating deaths avoided, life years gained and QALYs gained. Each of these outcomes are a function of the costs, life years and QALYs associated with continuing and stopping smoking and the impact of each package on the rate of cessation. These metrics were selected on the basis of previous work carried out by NICE and feedback from stakeholders 5, 7.

In forecasting outcomes such as life years, QALYs and costs, the model adopts a health‐care perspective in which costs and benefits to the health‐care system realized both by individuals who continue smoking and those who cease smoking within 1 year are estimated. The model has been designed to enable customization within the interface to provide estimates of costs and outcomes at various time‐points, including 2, 5 and 10 years and a life‐time (until an individual is 100 years of age). The model adopts country‐specific discounting rates 8, 9, 10, 11, 12.

### Design of the model

**Figure 1:**
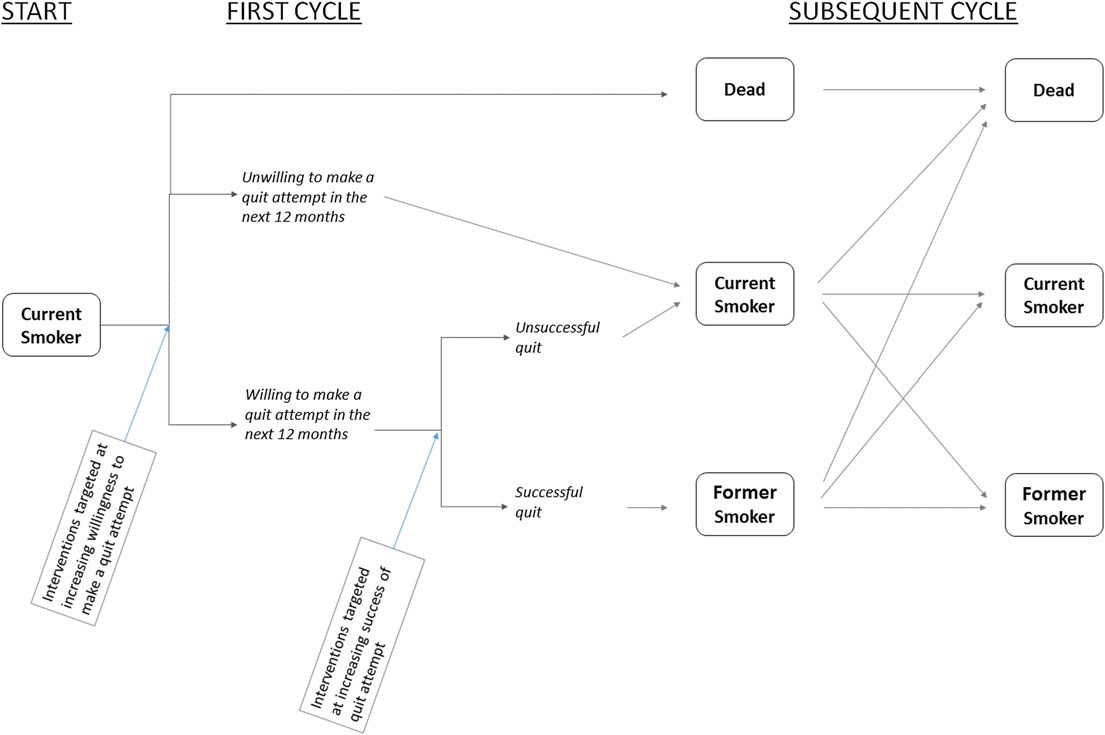
Schematic of Markov model [Colour figure can be viewed at http://wileyonlinelibrary.com]

The EQUIPT ROI tool uses a Markov state transition cohort model to estimate long‐term outcomes 13. There are three primary states within the model: current smoker, former smoker and death (Fig. 1). The cycle length is 1 year. The Markov model is constructed within Excel with a user‐friendly interface developed through Visual Basic.

With each 1‐year cycle the cohort is subjected to a set of transition probabilities which allow them to either stay within their current state or move to one of the other two states. Death is an absorbing state, meaning that those who enter this state remain within the state.

The model is replicated through a series of data tables within Excel for different population cohorts based on age (16–85; 18–85 years for Germany) and sex. To obtain population‐level estimates, these cohort‐level estimates are weighted by the percentage of the smoking population falling into each age (by individual year) and sex cohort 14, 15, 16, 17, 18, 19.

To calculate the relevant outcomes for a package of interventions, two separate models were created to simulate the health impacts of either quitting or not quitting smoking during the first cycle of the model— i.e. the cohort of smokers who quit and those who do not quit are modelled separately, with the results combined by weighting the outputs of the models by the country‐specific population and the package effectiveness and uptake.

### Transition probabilities

Within the model, three sets of annual transition probabilities are required:

- Package‐specific probability of quitting smoking in the first cycle (year) based on the uptake and effectiveness of interventions within the package;
- Age–gender‐specific annual probability of mortality associated with current smoking and being a former smoker; and
- Long‐term annual probabilities of smoking cessation for current smokers and relapse for former smokers.

The probability that current smokers will quit during the first cycle is a function of the proportion of the cohort who will make a quit attempt during the first year; the uptake of smoking cessation interventions in this group; the probability of a successful quit attempt with each intervention; and the probability of a successful quit attempt in those attempting to quit unassisted.

In order to estimate the probability of death by age and sex and smoking status, the following data were required: age–sex‐specific probability of death in the general population, the age–sex‐specific prevalence of smoking status (current, former or never smokers) and the relative risk of death by smoking status. Actuarial life tables and age–sex‐specific prevalence of smoking were provided by each of the participating countries 20, 21, 22, 23, 24, 25, 26, 27, 28, 29. The relative risks of death for former and current smokers versus never smokers were derived from published data 30.

Long‐term cessation and relapse were modelled through the application of an underlying quit rate which applies to all age–sex‐specific cohorts after the first year. The underlying quit rate represents a balance of those who quit smoking each year and those who start or relapse to smoking 31. For all participating countries this produces an underlying quit rate of approximately 2% in the general smoking population, except for within Hungary, where the rate is 1%.

### Prevalence of smoking‐related diseases

The Markov model estimates the proportion of the cohort who are either current smokers, former smokers or dead within each cycle. Based on available epidemiological data the model estimates, for each cycle, the prevalence of smoking‐related diseases through assessing smoking‐related population‐attributable fractions relating to current and former smokers.

Four diseases were incorporated, based on the increased burden associated with smoking behaviours: lung cancer, coronary heart disease (CHD), chronic obstructive pulmonary disease (COPD) and stroke 2, 32. These diseases account for approximately 75% of smoking‐related deaths 32. To estimate the number of cases each year of these diseases with and without smoking cessation during the first cycle, the following data were required: the prevalence of smoking‐related diseases, the prevalence of smoking status (current, former or never smokers) and the relative effect of smoking on the prevalence of each disease. Each partner country provided age–sex‐specific estimates of the first two parameters, while the same data are employed relating to relative effects 19, 23, 28, 33, 34, 35, 36, 37, 38, 39, 40, 41, 42, 43, 44.

### Costs

Within the model, costs include both the costs of the interventions contained within each package and the health‐care costs associated with these diseases. For each country, the annual health‐care costs for an individual with each of the diseases were estimated based on a systematic literature review 45, 46, 47, 48, 49, 50, 51, 52, 53, 54, 55. Costs relate to prevalent cases of disease. Based on the available data, estimates were derived either by dividing life‐time health‐care costs of disease by the estimated life expectancy after developing the disease or by dividing the estimated annual health‐care costs of the disease by the prevalence of the disease in the given year.

### Utility values

To identify sources for utility values for the model, each country conducted a search of the literature, with potential sources being evaluated against a predetermined criteria hierarchy. The criteria included the use of a validated instrument to evaluate quality of life, values reflective of the population of individuals with the disease/smoking status (i.e. the severity of disease and age/sex distribution of the sample from which the values were derived should be reflective of the population at large with the disease/smoking status), country‐specific sources and the incorporation of an estimate of the uncertainty in the reported value. Utility values based on current smoking status (current or former) were derived from the Health Survey for England adjusted for relevant covariates, including disease prevalence 56. Utility decrements associated with smoking‐attributable diseases were derived from the US Medical Expenditure Panel Survey 57. Both sets of utility values were elicited using the EQ‐5D questionnaire, a validated instrument for measuring health‐related quality of life, and UK tariffs were used for calculating utility scores. These two sources were selected as they met the greatest number of the criteria against which they were evaluated (i.e. three of the four requirements).

### Intervention‐based parameters

For each intervention a decision‐maker wishes to include within the model, the following data would be required: relative effect sizes, costs and uptake rate. Currently, the EQUIPT ROI tool incorporates such parameters for a variety of interventions.

### Obtaining parameter values

A comprehensive list of all parameters required within the model was provided to country‐specific modellers in order to facilitate the gathering of this information (Table 1). Modellers were required to search both administrative databases and published literature to source these data, which were then incorporated within the EQUIPT tool. The modellers completed this search and provided data to the principal modeller and the data were then incorporated within the current model.

**Table 1:** Data elements required to derive estimates of life‐time costs and quality adjusted life years (QALYs).

Parameter	Source
Discount rate	8–12
Population estimates by age and gender	14–19
Annual probability of death by age and sex	20–24
Prevalence of smoking by age and gender	25–29
Never smoker	
Former smoker	
Current smoker	
Relative risk of death by smoking status	30
Background quit rate	31
Relative risk of smoking attributable disease by age, sex and smoking status	2,32
Lung cancer	
CHD	
Stroke	
COPD	
Prevalence of smoking attributable disease by age and sex	19, 23,33–44
Lung cancer	
CHD	
Stroke	
COPD	
Costs of smoking attributable disease	45–55
Lung cancer	
CHD	
COPD	
Stroke	
Utility values by smoking status	56
Smoker	
Former smoker	
Never smoker	
Utility values associated with smoking attributable disease	57
Lung cancer	
CHD	
COPD	
Stroke	

CHD = coronary heart disease; COPD = chronic obstructive pulmonary disorder.

As the model is designed to give results over the entire smoking population and not the individual age–sex cohorts and is able to produce both country‐level and regional results, a number of additional input parameters with respect to population counts and vital statistics were required for populating the model. These included the population by age and sex, the prevalence of smokers and former smokers and the employment rate for smokers, all at both country and regional levels. Also required were country‐specific life tables for calculating mortality and information regarding inflation rates for adjusting historical costs and currency conversion rates.

### Key assumptions

As is the case with all models, a number of assumptions were required to estimate the economic impact of tobacco control interventions. These are described below.

The population‐based mortality rates are adjusted using the relative risks of death in smokers and former smokers, which are derived from the literature 30. Although the reference is dated and absolute mortality may have changed, the assumption that the relative effect was likely to be maintained and the choice of study was justified based on the prospective nature of the study, the sample size (*n* = 34 439) and the years of follow‐up (40 years).

The current model does not adjust explicitly for the time since quitting, due to the absence of distributional data regarding time since quitting and duration of smoking and the risk of smoking‐related disease and mortality. Rather, an average risk of smoking‐attributable disease and mortality is applied to former smokers. As this is a cohort, rather than an individual patient simulation model, the impact of this assumption may be limited.

Given the lack of data to support an alternative assumption, the prevalence of each disease is assumed to be independent of the prevalence of other diseases. Also, the model assumes that in the case of multiple diseases, the disutility associated with the disease with the greatest disutility is applied. This is a conservative approach, in that it provides a lesser estimate of the QALY gains from smoking cessation than either a multiplicative or additive approach.

For all diseases, in people aged below 35 years the risk of smoking‐attributable disease was assumed to be equal across smoking groups. This was deemed to be the most appropriate assumption, given that data regarding the differential rate of all diseases by smoking status were not available for this age group. This assumption is both conservative and, given the very low prevalence of disease in this group, unlikely to have a significant impact on the results.

The underlying quit rate, which applies to all cohorts after the first year, represents a balance of those who quit smoking each year and those who start or relapse to smoking. This assumption is supported by a meta‐analysis which showed that there was no difference in relapse rates after 12 months, regardless of whether the patients used an intervention to quit smoking or no intervention 58.

### Handling uncertainty

Within EQUIPTMOD, the effect of parameter uncertainty on the calculated outcomes can be assessed through probabilistic sensitivity analyses involving Monte Carlo simulation (MCS) 59. For the MCS, probability distributions related to natural history parameters, relative risks and odds ratios, costs and utilities are incorporated into the model.

Analysis adopts standard methods for defining uncertainty around parameters 59. Transition probabilities are characterized by beta distributions and relative risks and odds ratios are characterized by log‐normal distributions. Utility values by smoking status are characterized by beta distributions, while utility decrements associated with smoking‐related disease are characterized by normal distributions. Costs are characterized by gamma distributions. Both intervention costs and population level data are assumed fixed.

As a default, 1000 replications are conducted; i.e. a set of 1000 outcome estimates are obtained. The results are displayed by a scatterplot of costs versus QALYs and by cost‐effectiveness acceptability curves (CEACs) that present graphically the probability of being cost‐effective as a function of decision‐makers’ willingness to pay for a QALY 59.

### Analysis

EQUIPTMOD allows calculation of the costs, QALYs and life expectancy for a population of current smokers who quit smoking within the first cycle of the model and for a population of current smokers who do not quit smoking.

For illustration within this paper, the average costs, QALYs and life expectancy are presented as estimated for the smoking population in England, Germany, Hungary, the Netherlands and Spain. Discounted results are presented using country‐specific discount rates for a life‐time horizon. In addition, the discounted costs and QALYS over a life‐time horizon for England are presented by each age–sex strata and the discounted and undiscounted costs, QALYs and life years are presented for alternate time horizons for England.

Finally, illustrative examples of how these figures can be used are provided with respect to three specific forms of interventions for England—a population‐level intervention designed to increase the proportion of individuals who will make a quit attempt during the next 12 months; a novel individual‐based intervention designed to increase the quit rate in smokers making a quit attempt; and a population‐level intervention designed to increase the uptake of a cessation intervention in smokers willing to make a quit attempt. These analyses are conducted in the form of a cost–utility analysis.

## Results

### Estimated costs, life years and QALYs

Table 2 demonstrates the differences in life‐time outcomes between individuals who quit smoking during the first 12 months compared to those who do not. Over the life‐time horizon, quitting smoking during the next 12 months leads to an increase in QALYs of 1.09 (discounted), an increase in life expectancy of 0.66 (discounted) and a reduction in health‐care costs of £4961 (discounted). The magnitude of differences varied by countries, although the general trend in findings was maintained.

**Table 2:** Estimates of average costs, life years (LYs) and quality‐adjusted life years (QALYs) for life‐time horizons by whether individual stops smoking during the first cycle.

		England	Netherlands	Germany	Spain	Hungary
If individual quits in the next 12 months	Health‐care costs	£9602	€12 991	€11 838	€20 044	€10 775
	QALYs	15.86	16.18	14.81	16.14	14.40
	LYs	19.11	19.26	17.98	19.40	17.28
If individual does not quit in the next 12 months	Health‐care costs	£14 563	€20 045	€19 138	€37 742	€19 412
	QALYs	14.77	15.14	13.27	14.95	12.95
	LYs	18.45	18.26	16.75	18.78	16.28

All figures are discounted. Costs are presented in 2016 £ or €s.

Table 3 provides data for England based on alternative time horizons and by whether or not results are subject to discounting. There are no differences in life expectancy, either discounted or undiscounted, at 2 years, but by 5 years differences are observable with the difference over a life‐time horizon being 0.66 years (discounted) and 1.73 years (undiscounted). Differences in QALYs are observable at 2 years due to the improved utility value for former smokers compared to current smokers with a difference of 0.07 QALYs, both discounted and undiscounted. Over the life‐time horizon the QALY gains from quitting smoking are 1.09 QALYs (discounted) and 2.37 QALYs (undiscounted). For costs, differences are also apparent by 2 years. The costs for individuals who quit smoking during the first 12 months compared to those who do not were £519 lower (discounted) and £530 (undiscounted). Over the life‐time horizon the cost difference rose to £4961 (discounted) and £9225 (undiscounted).

**Table 3:** Estimate of average life years, quality adjusted life years (QALYs) and costs by alternative time horizons for England.

	Discounted			Undiscounted		
	Individuals who quit smoking in the 1st cycle	Individuals who do not quit smoking in the 1st cycle	Difference between quitters versus non‐quitters	Individuals who quit smoking in the 1st cycle	Individuals who do not quit smoking in the 1st cycle	Difference between quitters versus non‐quitters
Average costs						
2 years	£566	£1087	–£521	£576	£1106	–£530
5 years	£1409	£2635	–£1226	£1510	£2822	–£1312
10 years	£2764	£4966	–£2202	£3236	£5786	–£2550
Life‐time	£9.602	£14 563	–£4961	£23 486	£32 711	–£9225
Life years						
2 years	1.94	1.94	0.00	1.97	1.97	0.00
5 years	4.56	4.53	0.03	4.87	4.84	0.03
10 years	8.20	8.10	0.10	9.51	9.38	0.13
Life‐time	19.11	18.45	0.66	36.50	34.77	1.73
QALYs						
2 years	1.64	1.57	0.07	1.67	1.60	0.07
5 years	3.85	3.67	0.18	4.11	3.92	0.19
10 years	6.91	6.56	0.35	8.00	7.59	0.41
Life‐time	15.86	14.77	1.09	29.91	27.54	2.37

Figures are obtained by weighting estimates for each age–sex cohort for England by the proportion of the population in the cohort.

**Figure 2:**
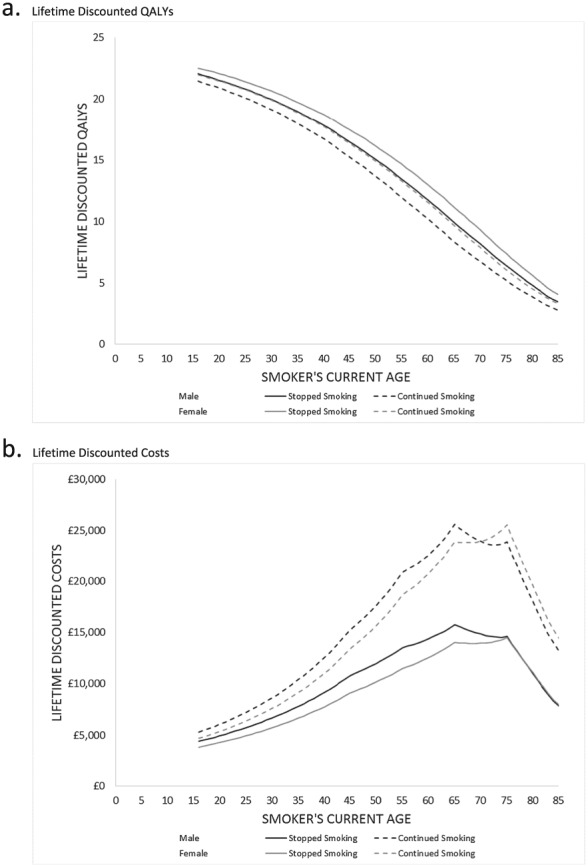
Life‐time costs and quality‐adjusted life years (QALYs) by whether or not individual stops smoking during the first cycle (England)

The absolute life‐time costs and QALYs by age and gender and smoking status for England vary, but the following findings were consistent (Fig. 2). For each age–sex category, costs were lower and QALYs higher for those who quit smoking during the first 12 months than those who did not. Life‐time QALYs tended to be higher for females than males within each smoking category. However, QALYs for males who quit smoking were higher than for females who did not quit smoking.

### Illustrative examples

For all the illustrative analyses, the following assumptions were made. The analyses were conducted for a smoking population of 2000. Analysis was conducted for a population corresponding to the current smoking population in England in terms of the age–sex breakdown and life‐time costs and QALYs. The proportion of smokers in the population who will make a quit attempt without any interventions will be 20%, and the success rate of an unaided quit attempt (i.e. without interventions aimed to increase the success rate of a quit attempt) is 5%.

For the first illustrative example, we will assume that there is a population‐level intervention that will lead to a relative increase in the proportion of smokers who will make a quit attempt of 25%. The population‐level intervention will cost £40 000, or £20 per smoker. Based on the estimated costs and QALYs in Table 2, the costs and QALYs associated with the two alternatives, the *status quo* and the addition of the population‐level intervention, can be estimated (Table 4). The incremental cost per QALY gained for the addition of the population level intervention is £2788, suggesting that the intervention is cost‐effective.

**Table 4:**
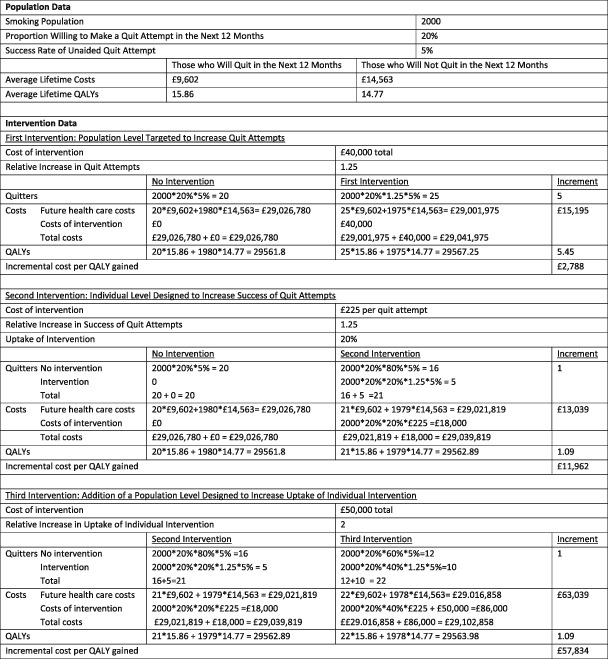
Results of illustrative examples.

For the second illustrative example, we will assume that there is a novel individual‐level intervention available for smokers making a quit attempt. The relative risk of quitting with the new intervention compared to an unaided quit attempt is 1.25, and the intervention will cost £225 per attempt. It is assumed that 20% of smokers making a quit attempt will utilize the new intervention. The incremental cost per QALY gained for the addition of the novel individual level intervention is £11 962, suggesting that the intervention is cost‐effective (Table 3).

For the third illustrative example, we will assume that there will be population‐level intervention to increase uptake of the novel individual‐level intervention described above. If the population‐level intervention is adopted, the proportion of smokers making a quit attempt who will use the novel individual‐level intervention will double. The population‐level intervention will cost £50 000. The incremental cost per QALY gained for the addition of the population‐level intervention compared to the novel individual‐level intervention is £57 834, suggesting that the intervention is not cost‐effective (Table 3).

## Discussion

In this paper we provide the background, methodology, limitations and initial results relating to EQUIPTMOD: the Markov model designed to facilitate the assessment of the cost‐effectiveness of alternative strategies to increase the rates of smoking cessation within participating countries. When designing studies to be transferable across jurisdictions, it is necessary to harmonize both the study/model design and data collection 60. The design of EQUIPTMOD facilitates such decisions not only in the countries participating in the first wave, as highlighted in this paper, but will allow countries with fewer analytical resources to adapt the model to provide pertinent information for their context.

EQUIPT is a rare multi‐disciplinary study which allows assessment of the transferability of economic evidence in tobacco control, and will provide an evidence‐based, practical and customizable ROI tool to actual decision‐makers. The findings are expected to promote and disseminate the ROI methods and results to foster evidence‐driven decision‐making on comprehensive tobacco control in Europe. By adopting a core methodological design but facilitating the incorporation of jurisdiction‐specific analytical features and data, EQUIPTMOD will be adjustable to fit the specific decision problem for multiple users.

There are limitations to the approach we have adopted. A major component of the model framework is the use of an underlying quit rate. The underlying quit rate has been estimated over time as the decrease in the percentage of the population who are smokers. This estimate has been consistent, and represents the impact of both quitting and relapsing. Similar data on the individual components are not readily available. As the model works at a population‐level, the use of an underlying quit rate is argued to be a reasonable compromise.

Analysis is based on a health‐care system perspective. Therefore, costs of social care associated with the diseases modelled are excluded. While this may be appropriate for economic evaluation, given the relevant guidelines, further analysis including social care costs could be conducted within a scenario analysis.

Within health economics, there is debate over the relevance of including health‐care costs that are unrelated to the specific context of interest but occur due to an intervention's impact in extending life. Guidelines typically suggest exclusion of such costs, but allow consideration of unrelated cost within scenario analysis. In the context of smoking cessation, such unrelated costs would occur only in the future. Furthermore, it would only include health‐care costs exclusive of heart disease, stroke, COPD and lung cancer, which would be both much smaller than all health‐care costs and difficult to measure. A further point is that annual health‐care costs for an individual are typically highest in the year prior to death. By delaying death through smoking cessation such high health‐care costs are not avoided, but are merely postponed to a later date. While inclusion of unrelated costs might increase the costs of quitters versus non‐quitters due to their longer life expectancy, the impact when assessing the cost‐effectiveness of smoking cessation‐related interventions compared to other uses of health‐care resources will be minimal. Inclusion of unrelated costs would lead to the incremental cost per QALY gained for all treatments which extend life to be increased by a similar factor—the annual costs of treating unrelated diseases. If the threshold value of a QALY is representative of the marginal costs of producing an additional QALY exclusive of unrelated costs, then inclusion of unrelated costs would simply lead to an increase in the appropriate threshold. Therefore, inclusion of unrelated costs is likely to have a minimal impact on decisions related to cost‐effectiveness. Given both this and the guidance on costing such studies, we did not include unrelated health or social care costs.

The development of EQUIPTMOD has several strengths. The decisions relating to model design and data assessment were made with all constituent partners having an equal voice with decisions based on consensus. The ‘inverted cone’ approach to the overall EQUIPT study has led to the development of a model which meets the specific needs for the initial study partners, but has the flexibility to meet the further needs of additional partners 6. Intensive stakeholder engagement throughout the research process highlights the design to be highly relevant to end‐users of research findings.

EQUIPTMOD has some limitations. Country‐specific modelling requires that data are available for each constituent country. However, extensive work within the EQUIPT transferability framework has allowed identification of those key data parameters for which country‐specific data are essential, allowing countries with fewer resources to target the acquisition of the most pertinent data elements.

The initial results highlighted in this paper indicate the large potential benefits from smoking cessation both at individual and population levels. Previous studies have highlighted that interventions designed to assist smoking cessation are consistently cost‐effective (e.g. 61, 62, 63, 64). EQUIPTMOD will be a further tool available to decision‐makers to assess the cost‐effectiveness of such interventions. However, given the multi‐faceted approach necessary to promote smoking cessation, EQUIPTMOD's greater application will be as the engine for the EQUIPT ROI tool to facilitate assessment by policymakers of the desirability of alternative packages of smoking cessation interventions.

Given the substantive role possible with the EQUIPT ROI tool and the potential applications of the underlying EQUIPTMOD economic model, it is hoped that the model described in this paper will be used frequently to assess both the cost‐effectiveness of individual interventions and to more generally foster evidence‐driven decision‐making on comprehensive tobacco control in Europe and beyond.

## Funding

The funding was received from the European Community's Seventh Framework Programme under grant agreement no. 602270 (EQUIPT).

## Ethics approval

None required for this study. However, the EQUIPT project was approved by Brunel University Research Ethics Committee.

## Data sharing

The model and accompanying documents are freely available to download from http://equipt.eu.

## Declaration of interests

R.W. undertakes consultancy and research for and receives travel funds and hospitality from manufacturing of smoking cessation medications but does not, and will not, take funds from e‐cigarette manufacturers or the tobacco industry. R.W. is an honorary co‐director of the National Centre for Smoking Cessation and Training and a Trustee of the stop‐smoking charity, QUIT. R.W.'s salary is funded by Cancer Research UK.
